# Ribosomal Protein Mutations Result in Constitutive p53 Protein Degradation through Impairment of the AKT Pathway

**DOI:** 10.1371/journal.pgen.1005326

**Published:** 2015-07-01

**Authors:** Ana T. Antunes, Yvonne J. Goos, Tamara C. Pereboom, Dorien Hermkens, Marcin W. Wlodarski, Lydie Da Costa, Alyson W. MacInnes

**Affiliations:** 1 Hubrecht Institute, KNAW and University Medical Center Utrecht, Utrecht, the Netherlands; 2 Department of Pediatric Hematology and Oncology, University of Freiburg, Freiburg, Germany; 3 AP-HP, Service d’Hématologie Biologique, Hôpital Robert Debré, Paris F-75019, France; 4 Laboratoire d'excellence, GR-Ex, Paris, France; 5 Université Paris VII-Denis Diderot, Sorbonne Paris Cité, Paris F-75475, France; 6 U1149, CRB3, Paris, France; Stowers Institute for Medical Research, UNITED STATES

## Abstract

Mutations in ribosomal protein (RP) genes can result in the loss of erythrocyte progenitor cells and cause severe anemia. This is seen in patients with Diamond-Blackfan anemia (DBA), a pure red cell aplasia and bone marrow failure syndrome that is almost exclusively linked to RP gene haploinsufficiency. While the mechanisms underlying the cytopenia phenotype of patients with these mutations are not completely understood, it is believed that stabilization of the p53 tumor suppressor protein may induce apoptosis in the progenitor cells. In stark contrast, tumor cells from zebrafish with RP gene haploinsufficiency are unable to stabilize p53 even when exposed to acute DNA damage despite transcribing wild type *p53* normally. In this work we demonstrate that p53 has a limited role in eliciting the anemia phenotype of zebrafish models of DBA. In fact, we find that RP-deficient embryos exhibit the same normal *p53* transcription, absence of p53 protein, and impaired p53 response to DNA damage as RP haploinsufficient tumor cells. Recently we reported that RP mutations suppress activity of the AKT pathway, and we show here that this suppression results in proteasomal degradation of p53. By re-activating the AKT pathway or by inhibiting GSK-3, a downstream modifier that normally represses AKT signaling, we are able to restore the stabilization of p53. Our work indicates that the anemia phenotype of zebrafish models of DBA is dependent on factors other than p53, and may hold clinical significance for both DBA and the increasing number of cancers revealing spontaneous mutations in RP genes.

## Author Summary

The *p53* tumor suppressor is the most commonly mutated gene in human cancers. However, cancer cells exploit multiple mechanisms to silence the p53 pathway in addition to inactivation of the *p53* gene. We previously reported that one of these mechanisms is found in tumor cells with ribosomal protein (RP) gene mutations. These cells transcribe wild type p53 mRNA yet do not stabilize p53 protein when exposed to DNA damaging agents. In this work we demonstrate that this loss of p53 protein is due to its constitutive degradation. This degradation is due to impairment of the AKT pathway, which normal signals for p53 to stabilize when the DNA is damaged. By re-activating the AKT pathway in RP-mutant cells we are able to restore p53 stabilization and activity, which may hold clinical significance for cancer treatment.

## Introduction

The stabilization of the p53 tumor suppressor is a pivotal event in the programmed cell death response. Levels of p53 protein are normally kept very low through its physical association with the MDM2 protein, an E3 ubiquitin ligase that constitutively ubiquitinates p53 and targets it for proteasomal degradation [1]. Many kinds of cellular stress, including DNA damage and oncogene presence, activate different signaling pathways that result in the dissociation of p53 and MDM2. p53 then stabilizes and translocates to the nucleus where it targets genes that arrest the cell cycle and turn on DNA repair, or genes that induce apoptotic cell death if the damage is deemed irreparable [2].

The stabilization of p53 has been reported to trigger human bone marrow failures such as dyskeratosis congenita and Fanconi anemia [3,4]. While Fanconi anemia is predominantly linked to mutations in DNA repair enzymes, several genes found mutated in dyskeratosis congenita patients have a known role in the rRNA maturation steps of early ribosome biogenesis. The mutation of these latter genes in zebrafish stabilizes p53, as does the mutation of several other genes important for the processing of rRNA [5–7]. In human bone marrow failures syndromes linked to RP haploinsufficiency such as Diamond-Blackfan anemia (DBA) and 5q-myelodysplastic syndrome, the loss of hematopoietic progenitor CD34+ cells by p53-induced apoptosis is believed by some to be the major cause of cytopenia [8]. However, the contribution of p53-induced apoptosis specifically to the cytopenia phenotype remains controversial. Recent studies demonstrated that patient CD34+ hematopoietic progenitor cells carrying mutations in the most commonly mutated gene linked to DBA (*RPS19*) do not reveal any hallmarks of apoptosis as they are induced to differentiate into erythrocytes [9]. This work also showed that while the co-depletion of *p53* with *RPL11* in CD34+ cells reduced some apoptotic effects, it did not restore the proliferation capacity lost upon *RPL11* depletion alone. Therefore the contribution of p53 stabilization to the loss of erythrocytes in DBA patients is possibly less significant than previously thought.

In addition to being important for erythrocyte production, there also exist several reports indicating a role for RPs as tumor suppressors. Human patients with 5q-MDS or DBA are more predisposed to developing malignancies, both acute myeloid leukemia (AML) and solid tumors, respectively [10,11]. Importantly, the advent of exome sequencing has unveiled a surprising number of somatic RP gene mutations in an array of human cancers. These recent exome sequencing reports have identified mutations in *RPL5* and *RPL10* in T-cell acute lymphoblastic leukemia, mutations in *RPL5* in gliomas, and mutations in *RPL22* in human endometrioid endometrial cancer and colorectal cancer [12–14].

Embryonic zebrafish mutants and morphants are popular models of DBA and RP loss. In mutant lines where RP genes are disrupted by murine virus integrations, the homozygous embryos reveal a progressive reduction of the RP over the first 3 days post fertilization (dpf) coupled to a failure of hemoglobin-expressing cells to develop [15,16]. In contrast, haploinsufficient RP embryos reveal no cytopenia or any other conspicuous phenotype except for a mild growth defect that does not affect their development into adulthood [17]. Once reaching adulthood however, many of the haploinsufficient mutant lines reveal the formation of malignant peripheral nerve sheath tumors (MPNSTs), a tumor type rarely observed in laboratory strains of zebrafish [18]. Interestingly, this exact tumor type arises with the same frequency in zebrafish carrying homozygous mutations of *p53* in a highly conserved residue within one of the DNA-binding domains (M214K) [19]. Further study into this revealed that while wild type *p53* mRNA was normally transcribed in the RP-mutant tumor cells, p53 protein was unable to be detected [20]. This observation was despite the application of ionizing radiation and/or proteasome inhibition with MG132, two conditions that were found to stabilize p53 in zebrafish tumor cells carrying wild type RP genes.

Actinomycin D is a drug that disrupts ribosome biogenesis by inhibiting rRNA polymerase and results in the stabilization of p53 [21]. It was recently reported that this induction of p53 requires the activity of the survival factor AKT/PKB [22], a kinase that responds to the stimulation of many growth factor receptors, including insulin receptors [23]. While activated AKT has many functions, one major event of insulin stimulation directly downstream of AKT is the phosphorylation and inhibition of glycogen synthase kinase-3 (GSK-3) [24]. Under normal conditions GSK-3 phosphorylates and activates MDM2 in a way that promotes p53 degradation [25]. However upon DNA damage by ionizing radiation, the phosphorylation of AKT results in the inactivation of GSK-3 [26]. This AKT-mediated inactivation of GSK-3 in response to ionizing radiation begins with the DNA-dependent protein kinase (DNA-PK), a protein that recognizes the double-stranded breaks on DNA and signals through a cascade that ultimately results in p53 stabilization and programmed cell death [27]. Thus one mechanism of the p53 stabilization in response to ionizing radiation is through the activation of DNA-PK and AKT inhibiting the downstream activity of GSK-3 and MDM2.

We previously reported that the loss of RP genes in mammalian cells and in zebrafish embryos results in a loss of AKT activity that could be overcome by the addition of insulin [16]. This observation led us to consider the possibility that the AKT pathway was involved in the regulation of p53 in RP mutant cells, and that the impairment of the DNA damage pathway through AKT may have a role in the predisposition to malignancy caused by RP gene mutations.

## Results

### The hematopoietic phenotype of zebrafish with RP loss is not dependent on p53

The RP mutant zebrafish lines we used for this study were generated by viral insertions in the introns of RP genes, two of which (*rpS7* and *rpL11*) have homologues found mutated in DBA patients [28,29]. We find that at 2 dpf these embryos display normal expression of the *scl* transcription factor required for the genesis of hematopoietic stem cells in both primitive and definitive hematopoiesis, a result that was also observed in zebrafish embryos with deletions in the *rpS19* gene (S1 Fig) [30–33]. However, RP mutants reveal a marked decrease in the expression of the *βE1-globin* gene, which (at this stage of development) normally becomes up regulated as cells commit to the erythrocyte lineage (S1 Fig) [34]. Zebrafish embryos carry maternal stores of RPs such that these mutants reveal a progressive loss of RP expression. The *rpS7* mutants express about 50% of wild type rpS7 levels at 1 dpf while this percent reduction is not observed in *rpL11* mutants until 3 dpf [16,35]. This likely explains why despite the fact that embryos from both mutant lines reveal hematopoietic and developmental phenotypes, these are more severe in the *rpS7* mutants [16,35]. Because these phenotypes at 1 dpf are much more pronounced in *rpS7* embryos, we selected them for the following analysis of apoptosis. S2 Fig provides images of the mutants compared to wild types and shows the morphological features that we use to initially select the mutants, such as the smaller head and eye, inflated hindbrain, and the dent in the mid-hind brain barrier.

**Fig 1:**
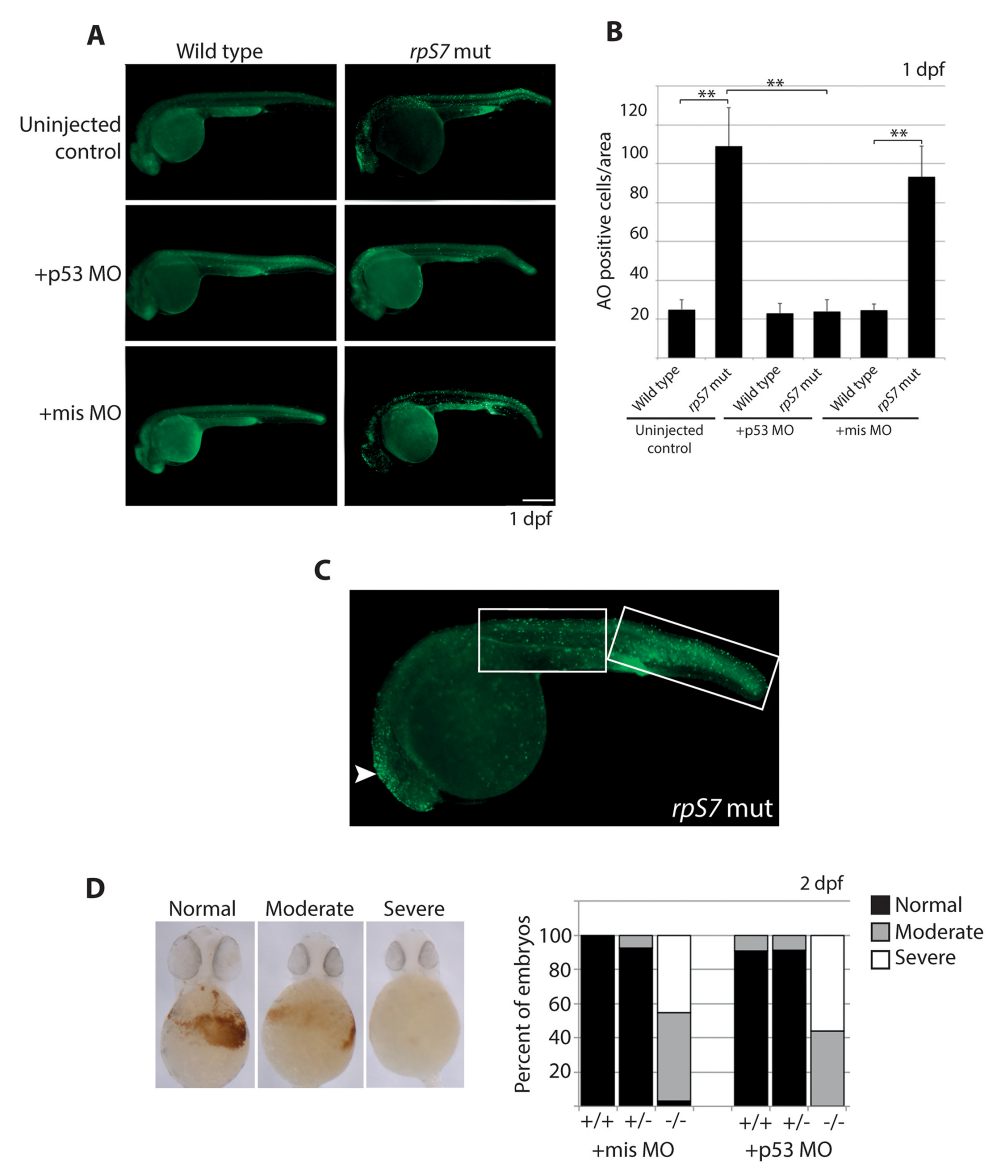
Early cell death of RP mutant cells requires p53. **A)** Acridine orange staining allowing visualization of dying cells in wild type or *rpS7* mutants at 1 dpf that are uninjected, injected with the p53 MO, or injected with the missense MO. Size bar = 0.25mm. **B)** Quantification of (A). **p<0.01. **C)** Acridine orange staining of a *rpS7* mutant is observed predominantly in the brain region (arrowhead) or distributed over the surface of the tail (white boxes). **D)** Scoring results from o-dianisidine staining allowing visualization of hemoglobin-expressing cells in clutches of either 111 embryos (+mis MO) or 177 embryos (+p53 MO) from an *rpS7* pairing.

We measured overall levels of cell death in developing embryos carrying the *rpS7* mutation with acridine orange (AO), a stain commonly used to detect cells undergoing apoptosis in zebrafish embryos [36]. Fig 1A and 1B show that at 1 dpf, *rpS7* mutant embryos reveal a significantly larger number of apoptotic cells compared to wild type controls. Closer visualization of an *rpS7* mutant in Fig 1C reveals that these AO-stained cells are found clustered in the brain area and evenly distributed on the entire surface of the tail. The injection of a translation-blocking morpholino that we have previously demonstrated is specific to zebrafish p53 (p53 MO) but not a missense control morpholino (mis MO), completely rescued the number of cells undergoing apoptosis in the *rpS7* mutants, reinforcing a role for p53 in cell death as a result of RP loss (Fig 1A and 1B) [37]. The p53 MO also results in the rescue of morphological phenotypes commonly observed in ribosome biogenesis mutants such as the inflation of the hindbrain vesicle and pericardial edemas, a rescue effect that has been previously demonstrated in other zebrafish models of RP loss (S2 Fig) [15,38]. To determine if the p53 MO was sufficient to rescue the defective hematopoiesis of the mutants we used o-dianisidine, which stains hemoglobin-expressing cells evident at 2 dpf. Staining with o-dianisidine revealed that despite the rescue of apoptotic cells by p53 depletion observed at 1 dpf in the *rpS7* mutants, the p53 MO was not able to rescue hematopoietic development to any appreciable degree (Fig 1D).

### The apoptotic response to DNA damage is caspase-independent in RP mutants

**Fig 2:**
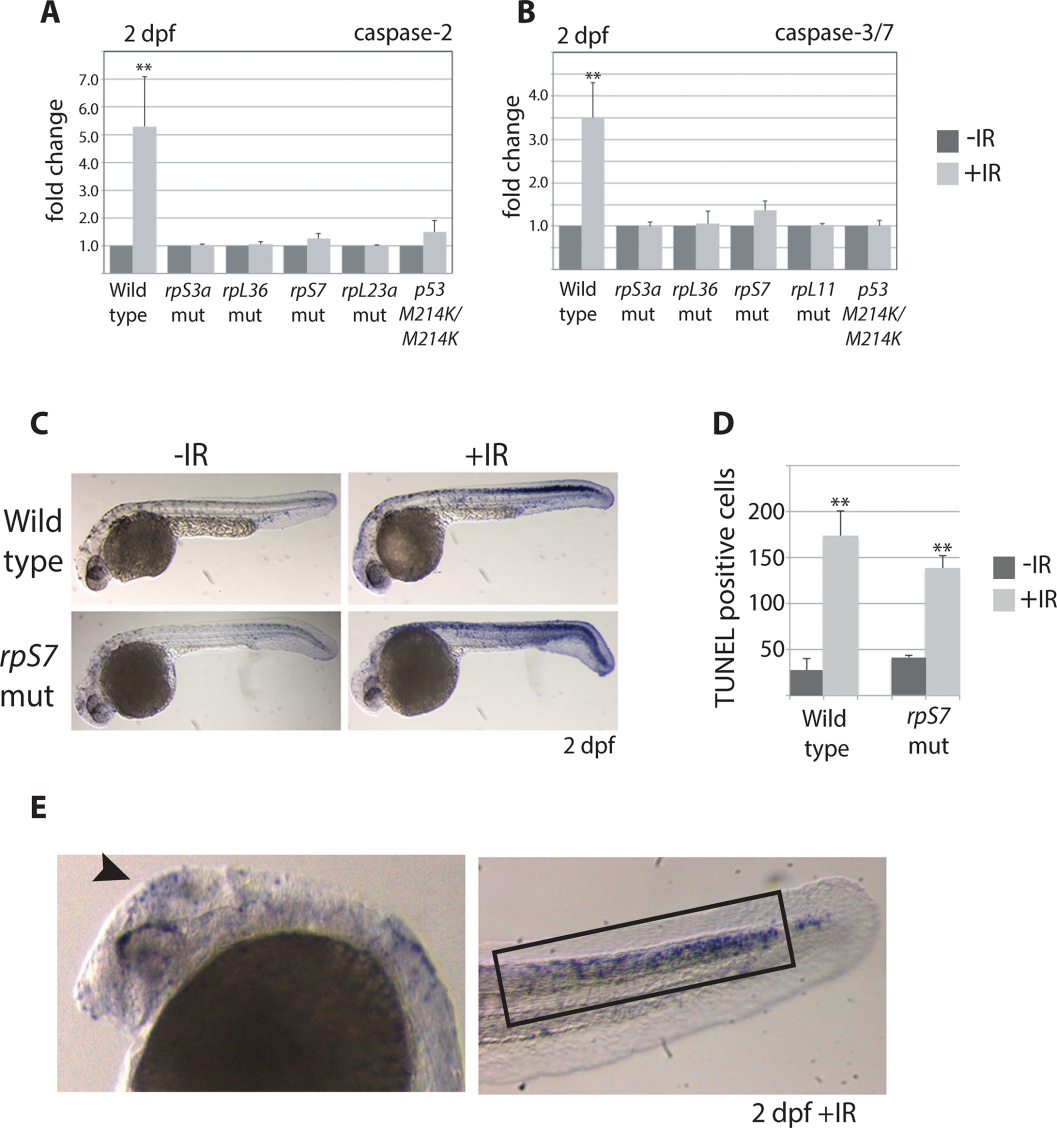
Caspase 3/7 activation is increasingly impaired in RP mutants. **A)** Caspase 2 and **B)** 3/7 activity is measured using a proluminescent caspase-3/7 DEVD-aminoluciferin substrate in 2 dpf embryos either untreated or exposed to 25 Gy ionizing radiation (IR). **C)** TUNEL analysis of 2 dpf wild type or *rpS7* embryos reveals cells with DNA fragmentation when embryos are exposed to 25 Gy IR. **D)** The number of TUNEL-positive cells in (**C**) are quantified. **E)** TUNEL-positive cells in both wild type and *rpS7* mutants are observed predominantly in the brain region (arrowhead) or in the tail tissue dorsal to the notochord (black box). *p<0.05, **p<0.01.

The levels of caspase-induced apoptosis in human CD34+ cells with RP loss vary depending on the RP [9]. To determine if the loss of RPs triggers caspase-induced apoptosis in zebrafish embryos we measured both the basal levels of activated caspase 2 or 3/7 and their levels in response to ionizing radiation, comparing wild type embryos with those carrying mutations in *rpS7* and *rpL11*, as well as *rpS3* and *rpL36* genes. Fig 2A and 2B indicate that in 2 dpf embryos the caspase 2 and 3/7 basal levels in the mutants is equivalent to wild types, and the caspase response to DNA damage is severely impaired in all the mutants. In fact, the RP mutant lines show the same suppressed caspase 2 and 3/7 response to ionizing radiation as the homozygous *p53* ^*M214K/M214K*^ mutant line (Fig 2A and 2B), which is severely impaired in its ability to induce apoptosis [19]. To determine if other hallmarks of apoptosis such as DNA fragmentation are present in RP mutants we performed TUNEL assays on *rpS7* embryos at 2 dpf. Fig 2C and 2D show that while there is no appreciable difference in the levels of TUNEL-positive cells between the *rpS7* mutant and wild type embryos, the exposure of mutants to ionizing radiation results in a similar significant increase of TUNEL-positive cells as observed in the wild types. Closer visualization of these DNA-damaged embryos reveals a localization of TUNEL-positive cells in the brain area similar to what is seen with the AO staining, however the localization of TUNEL-positive cells in the tail region is found much more restricted to the dorsal area above the notochord (Fig 2E). This may be due to the limits of penetration of the AO stain, or may suggest that the cells in this dorsal area are especially sensitive to DNA damage-induced apoptosis, as are the rapidly proliferating cells in the brain.

### p53 stabilization but not transcription is impaired in RP mutants

**Fig 3:**
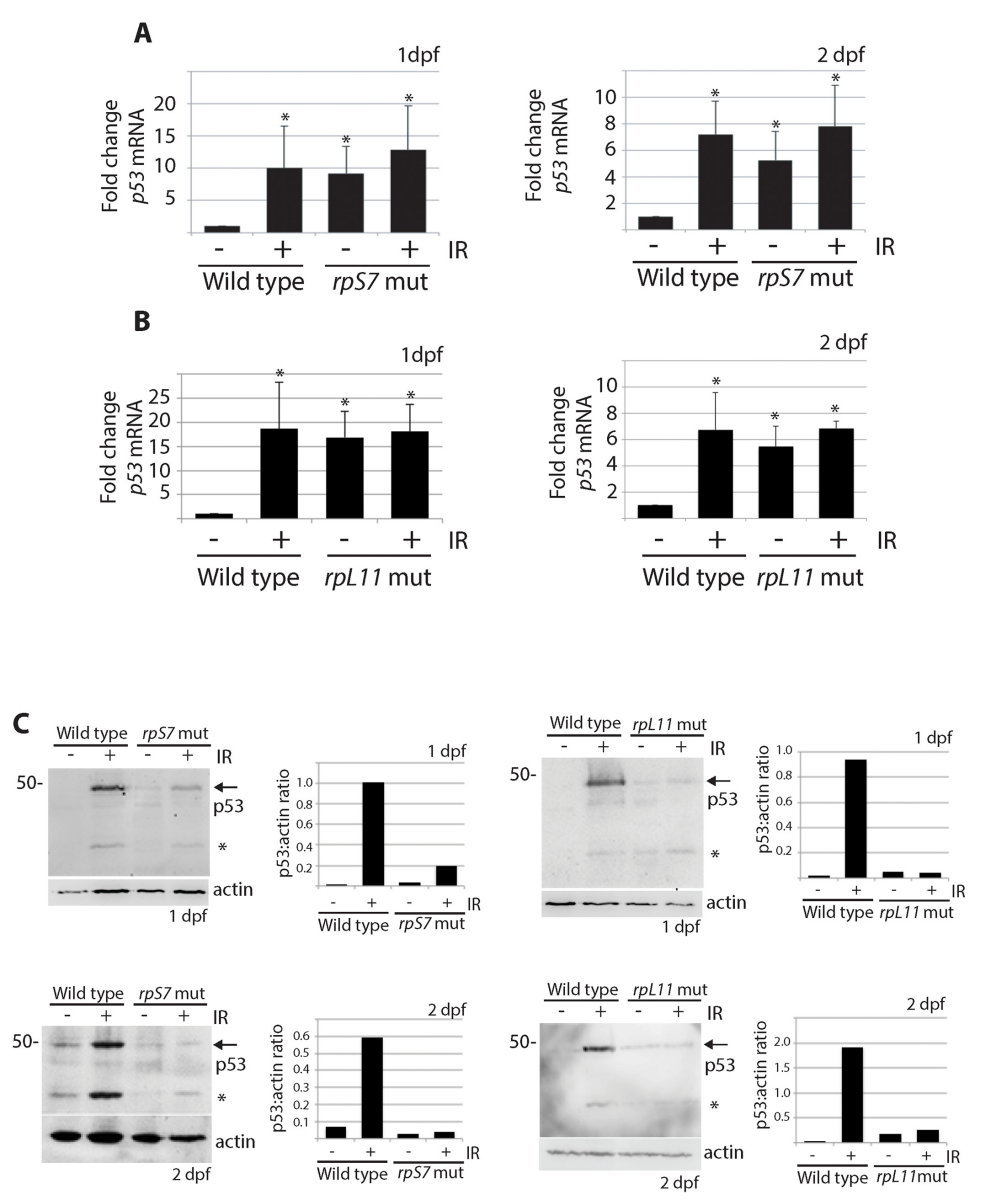
p53 protein stabilization is impaired independently of *p53* mRNA levels. **A)** qPCR analysis measuring levels of *p53* mRNA in wild type or *rpS7* mutants at 1 or 2 dpf either untreated or exposed to 25 Gy ionizing radiation. **B)** qPCR analysis of *p53* mRNA levels in wild type or *rpL11* mutants at 1 or 2 dpf either untreated or exposed to 25 Gy ionizing radiation. *p<0.05. **C)** Western blot analysis of p53 protein levels and the quantification of the p53:actin ratio of in *rpS7* or *rpL11* mutants at 1 or 2 dpf either untreated or exposed to 25 Gy ionizing radiation. * indicates either a p53-specific isoform or a degradation product.

The increased transcription of the zebrafish *p53* gene and its *p53Δ113* isoform (a target gene of stabilized p53) has been described in several models of RP loss and likely reflects the early response of p53 that triggers an up regulation of its own transcription and the transcription of *p53Δ113* [15,35,38–41]. In line with these results, we found using real-time quantitative PCR analysis with primers that amplify both full-length *p53* and *p53Δ113* that *p53* mRNA levels were significantly higher in *rpS7* and *rpL11* mutants at 1 and 2 dpf both in the presence and absence of ionizing radiation compared to untreated wild type embryos (Fig 3A and 3B). Semi-quantitative PCR analysis of *p53* mRNA levels in several other RP-mutant embryos (*rpS3a*, *rpL23a*, and *rpL36*) at 2 dpf similarly revealed equivalent levels of *p53* transcription in the mutants compared to wild types (S3 Fig). However, when we performed western blotting analysis using a zebrafish p53-specific antibody, we were unable to detect any appreciable amount of p53 protein in the *rpS7* or *rpL11* mutants in either the presence or absence of ionizing radiation at either 1 or 2 dpf (Fig 3C). This is the case in all the mutant RP lines we tested including in *rpS3a*, *rpL23a*, and *rpL36* (S3 Fig). We often observe what may be a p53-specific isoform such as *p53Δ113* on the western blots, but this may also be a p53 degradation product and in this work we cannot be certain of its exact identity. Taken together, the results suggest that although the p53 response to the RP mutation on a transcriptional level may function normally, an additional level of p53 post-translational regulation exists in the presence of RP mutations that serves to reduce p53 protein.

### p53 protein is degraded by the proteasome and rescued by insulin in RP mutants

**Fig 4:**
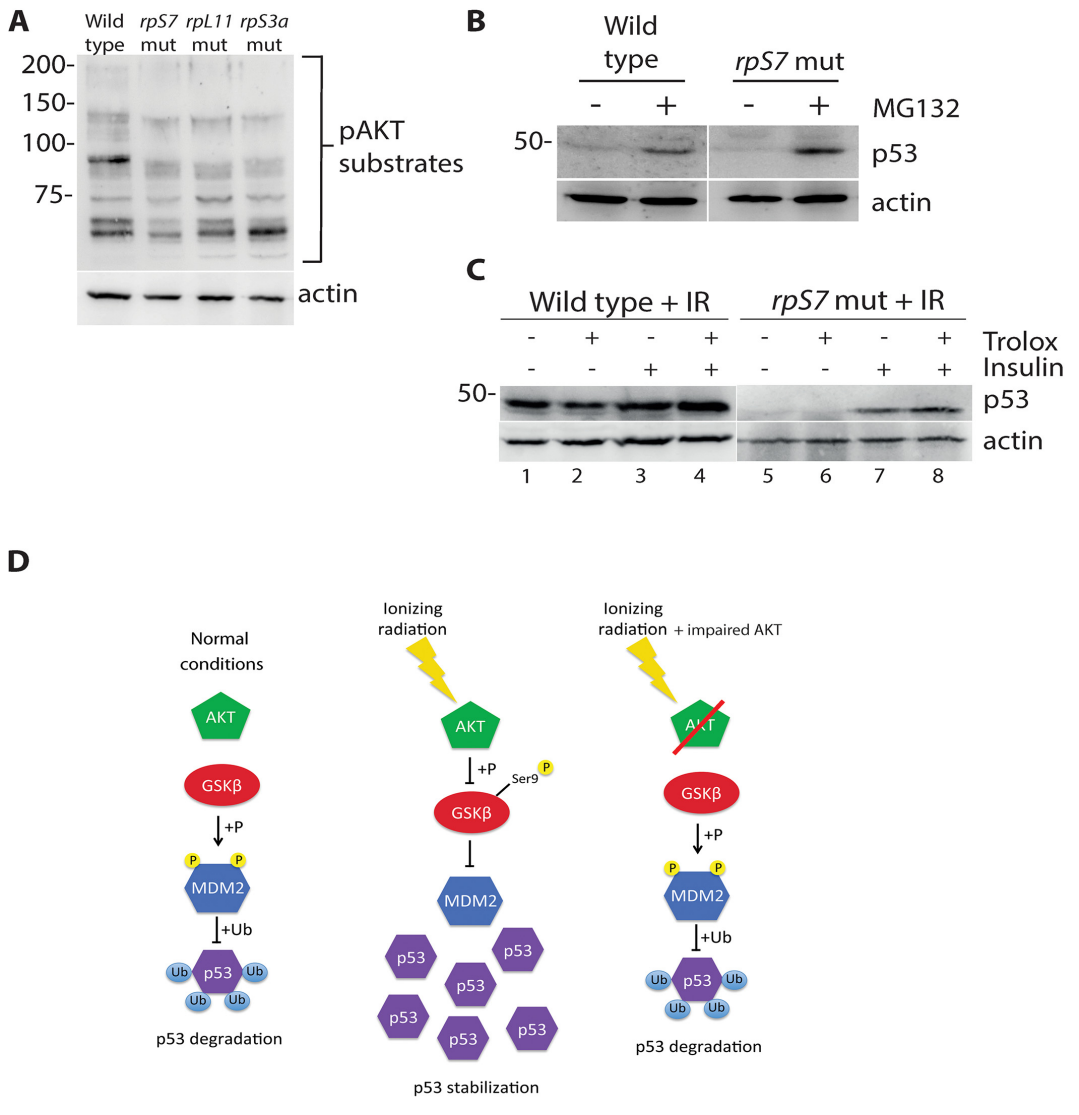
p53 protein is degraded by the proteasome and rescued by insulin in RP mutants. **A)** Western blot analysis of p53 levels in 2 dpf wild type or *rpS7* mutant cells untreated or exposed to 20μM of the proteasome inhibitor MG132. **B)** Western blot analysis of p53 protein levels in either wild type or *rpS7* mutants exposed to 25 Gy ionizing radiation followed by the addition of 350nM insulin and/or 10mM Trolox. **C)** Model illustrating how impaired AKT activity by RP mutations may result in the failure of p53 to stabilize in response to ionizing radiation.

We recently demonstrated that AKT phosphorylation activity is impaired in zebrafish embryos carrying mutations in *rpS7*, *rpL11*, and *rpS3a* [16]. Since the loss of AKT activity would theoretically lead to an increase of p53 proteasomal degradation due to an increase of GSK-3 phosphorylation of MDM2, we used the proteasome inhibitor MG132 to determine if we could restore p53 expression. Fig 4A illustrates that MG132 is able to stabilize p53 in wild type embryo cells as expected, and moreover it is able to stabilize p53 in the *rpS7* mutants. This supports the notion that the loss of p53 in the RP mutants is the result of excessive proteasomal degradation. We therefore hypothesized that stimulating AKT in the presence of ionizing radiation may rescue the p53 response to DNA damage. Fig 4B (lanes 1–4) shows p53 stabilization approximately 6 hours after exposure of wild type embryos to ionizing radiation when the embryos are treated with the anti-oxidant Trolox, insulin, or both. Fig 4B (lanes 5–8) illustrates that the addition of insulin, but not Trolox, immediately following the ionizing radiation results in a rescue of the stabilization of p53 in *rpS7* mutants. These data suggest that overcoming the RP mutation-induced inhibition of AKT with insulin, which would stimulate AKT more directly than Trolox, is able to rescue the p53 stabilization response to ionizing radiation. A diagram illustrating how the impairment of the AKT pathway can lead to p53 protein degradation through GSK-3 is shown in Fig 4C.

### GSK-3 inhibition restores p53 in RP mutant cells

**Fig 5:**
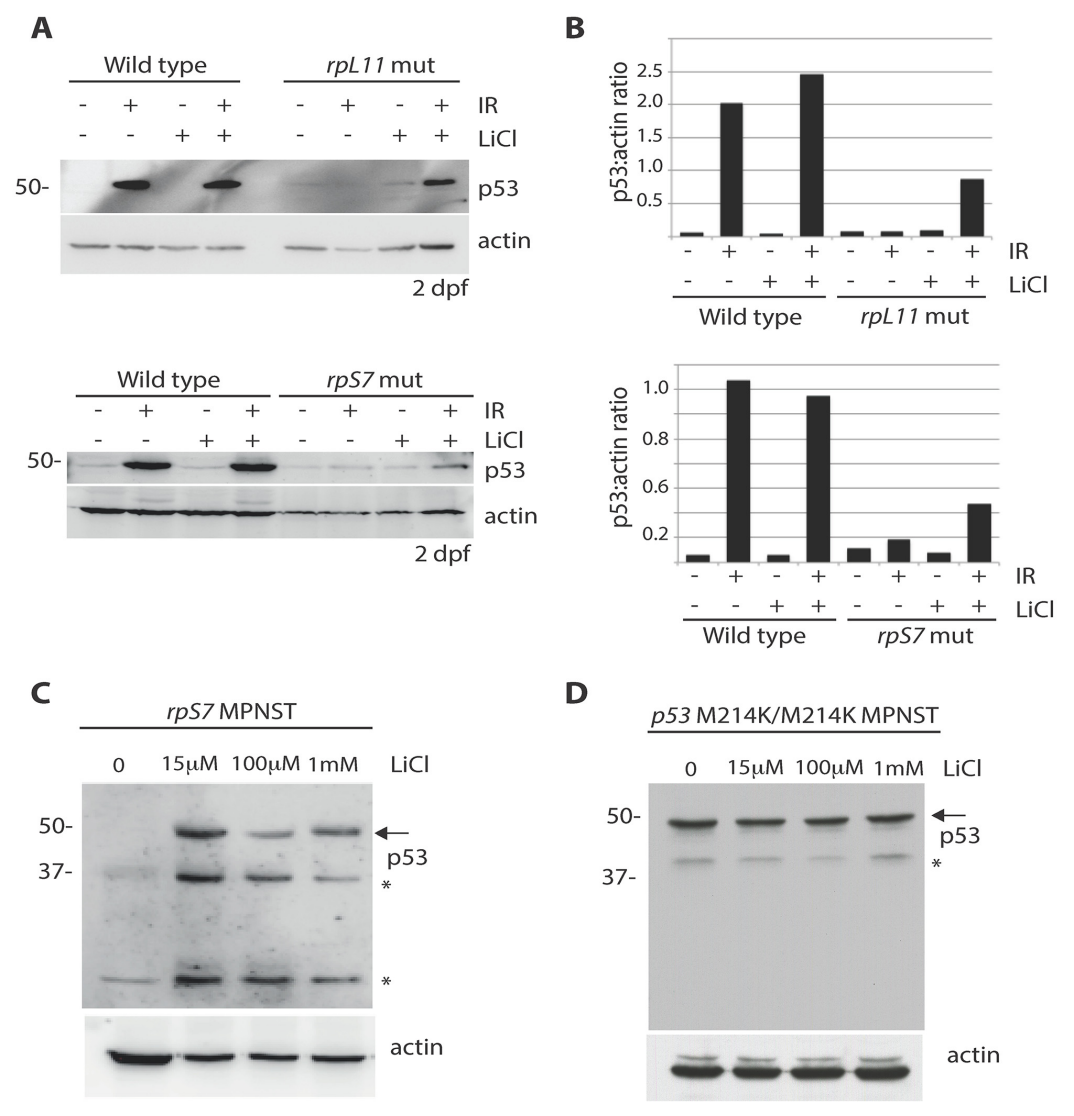
Lithium chloride restores p53 stabilization in RP mutant embryos and tumor cells. **A)** Western blot analysis of p53 levels in 2 dpf wild type, *rpS7*, or *rpL11* mutant embryos either untreated or exposed to 25 Gy ionizing radiation followed by the addition of 100μM LiCl. Note the partial rescue of p53 stabilization in the RP mutant embryos when exposed to LiCl after IR. **B)** Quantification of the p53:actin ratio of the western blots from (**A**). **C)** Western blot analysis of p53 levels in *rpS7* MPNST tumor cells untreated or incubated with varying concentrations of LiCl. **D)** Western blot analysis of p53 levels in *p53* ^*M214K/M214K*^ MPNST tumor cells untreated or incubated with varying concentrations of LiCl. * indicates either a p53-specific isoform or a degradation product.

We hypothesized that the impairment of AKT activity observed in cells with RP mutations could cause constitutive activation of GSK-3, phosphorylation of MDM2, and ultimately resulting in the constitutive degradation of p53. Therefore we reasoned that the inhibition of GSK-3 with lithium chloride (LiCl) in cells with RP mutations could restore p53 stabilization. Fig 5A and 5B show a partial rescue of p53 stabilization in *rpL11* and *rpS7* mutant embryos exposed to ionizing radiation followed by 6 hours of LiCl treatment. Finally, p53 expression is completely restored in *rpS7* haploinsufficient MPNST tumor cells when the cells are plated overnight with different dosages of LiCl (Fig 5C). This figure also shows LiCl induces the expression of what may be different isoforms of p53 such as p53Δ113 resulting from restored transcriptional activity of p53 in the tumor cells, for LiCl treatment of MPNST tumor cells expressing the transcriptionally impaired p53^M214K/M214K^ mutant does not result in the appearance of same bands (Fig 5D). However we are still unable to state for certain what the exact identity of these bands are, although they appear to be specific to p53 activation. These data strongly implicate the impairment of AKT, in particular the loss of AKT inhibition of GSK-3, as a driving force behind the high levels of constitutive degradation of p53 observed in tumor cells with RP gene haploinsufficiency.

## Discussion

While stabilization of the p53 tumor suppressor has long been held the culprit for the cytopenia phenotype observed in human diseases linked to RP gene mutations, it has been a decade-long mystery as to why no p53 protein is detectable in RP haploinsufficient tumor cells. In the context of the RP mutant zebrafish embryos, we show that a similar loss of p53 protein is evident as the embryos age to 2 dpf, the maternal stores of RPs are depleted, and the RP deficiency resulting from the mutation becomes more severe. We do not believe that this loss of p53 in RP mutants is simply due to the inability of the cells to make protein per se, for in other zebrafish models of ribosome biogenesis deficiency such as nucleostemin, *gnl2*, and *nop10* mutants we are able to visualize robust p53 stabilization by western blot analysis even in the absence of ionizing radiation up until 4 dpf [5,6]. Interestingly, the *nop10*, *gnl2*, and nucleostemin genes all code for proteins with important roles in early ribosome biogenesis and the processing of rRNA. While it has been shown that there are indeed detectable defects in rRNA processing in DBA patient CD34+ cells with RP mutations, these are by and large more subtle than the defects we observe upon the loss of *nop10*, *gnl2*, or nucleostemin [5,6,42]. This suggests that, as with actinomycin D, defective rRNA processing is a critical mediator of p53 stabilization and may in fact be a causative agent in the cytopenia phenotype of human diseases such as dyskeratosis congenita. However, our data suggest the molecular pathology underlying the anemia in diseases linked to RP mutations is likely to implicate mechanisms that go beyond stabilization of the p53 protein.

The fact that others and we observe no difference in early hematopoietic stem cell expression in RP mutant zebrafish embryos coupled to the anemia failing to be rescued upon p53 loss suggests that the anemia phenotype cannot be explained by the organism’s general p53 response to the ribosome biogenesis defects induced by RP loss [30]. Clearly the embryos younger than 1 dpf are experiencing some p53-induced apoptosis otherwise we would see no AO staining rescue upon injection of the p53 MO and no partial rescue of the morphological phenotypes. We therefore propose that p53 has such an early effect in the developing RP mutant embryo that by the time of 1 dpf (approximately 30 hours post fertilization is when the experiments would begin) all that we are able to observe by laboratory techniques are the apoptotic cells in the wake of a brief p53 activation. The remaining live cells at 1 dpf appear devoid of p53 protein, and if the apoptotic cells do still carry p53 protein we cannot detect it by western blotting. We were unable to detect p53 by western blotting in any embryo younger than 1 dpf, but this is likely a technical issue reflecting the abundance of yolk protein still present at this early stage. The apoptotic cells rescued by p53 loss that we are able to observe at 1 dpf also do not appear to be important for red cell development, as we detect no rescue of the anemia phenotype upon p53 loss.

The results of TUNEL assay suggests that some DNA fragmentation upon acute DNA damage is still possible in the 2 dpf RP mutant cells despite no evidence of p53 stabilization or caspase activation. Other studies have reported similar findings of caspase-independent DNA fragmentation in cancer epithelial cells in response to diverse toxins, and the authors of this work suggest that the DNA damage induced by these toxins may lead to noncaspase-mediated proteolytic activation of DNases [43]. Interestingly, studies in *Drosophila* have shown that ionizing radiation leads to cells acquiring RP haploinsufficiency, and that these cells undergo apoptosis that is also p53-independent [44]. Taken all together, our work supports a model where the impairment of mature red blood cell formation in organisms with RP deficiency is due to cell loss that is not dependent on either p53 stabilization or caspase activation. However, it should be pointed out here that the work in this study is entirely based on zebrafish models of DBA, which carry homozygous RP mutations (as noted in the introduction, the haploinsufficient mutants have no embryonic phenotype except a slight growth defect). Therefore it may be that p53 has a more prominent role in cells with true RP haploinsufficiency, and this remains an issue that must be kept in mind when interpreting data generated from zebrafish models of DBA.

While other studies have suggested that the p53 MO or *p53* ^*M214K/M214K*^ mutant background rescues both general apoptosis and the number of hemoglobin-expressing cells in embryos deficient for RPs [15,38], we believe our genetic models are more consistent than using morphants and that our approach to quantifying the mutant phenotypes are both more robust (we use sample sizes N > 100) and reliable (we genotype the embryos after the blind scoring to resolutely identify the homozygotes). Other studies support us by demonstrating that p53 independent pathways are contributing to the anemia phenotype of RP-mutant zebrafish embryos, and that the loss of p53 rescues the morphological abnormalities but not the anemia phenotype of embryos with reduced RP expression [45–47].

It remains an open question as to what the major pathways beyond p53 and caspase activation contribute to the death of RP deficient cells and the anemia phenotype in zebrafish models of DBA. Interestingly, a recent study has shown that the mutation of *rpS19* in zebrafish embryo erythrocytes specifically reduces the translation, but not the transcription of the hemoglobin gene *hbbe3* [30]. We also recently reported that the up regulation of autophagy, the cellular process of self-digestion that is tightly regulated during hematopoiesis, is observed in both zebrafish models of DBA and in human DBA cells with RP haploinsufficiency [16]. It may therefore be that RP loss in maturing erythrocytes derails their proper differentiation by failing to translate critical mRNAs, or by the constitutive activation of autophagy resulting in the erythrocyte progenitor cells essentially eating themselves before they are able to fully differentiate. The former possibility is supported by several findings suggesting that L-leucine, an amino acid that increases translation by activating the mTOR pathway, is able to partially rescue the anemia phenotype of zebrafish RP morphants and increases the number of erythroid cells in red cell culture assays where CD34+ cells are infected with shRNAs against *RPS19* or *RPS14* [47,48]. The latter possibility is enticing for one would not expect constitutively up regulated autophagy to elicit an apoptotic response. Or the cells may be undergoing an as of yet unknown p53- and caspase-independent mechanism of cell death that awaits identification.

We had previously reported that the addition of MG132 to the MPNST cells with RP gene haploinsufficiency was not able to restore p53 stabilization [20]. At the time this led us to believe that the block in p53 expression was at the level of protein synthesis, since MG132 should be able to stabilize any protein undergoing ubiquitination and proteasomal degradation. However our present study suggests otherwise, that in fact the MG132 treatment is not sufficient to restore p53 stabilization in RP mutant MPNST cells. There may be additional factors at the tumor level contributing to the degradation of p53 that are as of yet not known, or it may be that the robustness of the degradation in the tumors is much stronger than in the embryos. While our present study is not able to delineate between these possibilities, it may be that more powerful proteasome inhibitors are capable of restoring p53 to the same levels as we observe with the LiCl treatment of tumor cells, and these studies will be of great future interest. It should also be pointed out here that given the very wide range of effects that LiCl has on many cellular signaling pathways, there may be other effects beyond the AKT pathway that are contributing to the restoration of p53 that we observe.

Very recent work suggests that a mechanism for malignant transformation in RP-haploinsufficient cells involves cells acquiring oncogenic mutations that allow for the bypass of a 60S ribosomal subunit integrity checkpoint [49]. We propose that these mutations (which have yet to be identified) coupled to excessive degradation of wild type p53 would be sufficient to promote malignant transformation. Interestingly, the T-ALL cells with *RPL5* and *RPL10* mutations, similar to the RP-haploinsufficient zebrafish tumor cells, the *p53* gene remains wild type (personal communication with Dr. De Keersmaecker). We thus posit that cells with RP deficiencies are able to exert selective pressure to overcome the p53 activity not just by acquiring *p53* gene mutations but also by suppressing AKT activity.

DBA is a rare disease with a prevalence that is approximately 7 in 1 million live births [50]. In addition, since the majority of DBA patients die early in life from bone marrow failure or from complications stemming from chronic blood transfusions, the number of DBA patients who ultimately experience malignant transformation is very low (it would be next to impossible for us to obtain DBA patient-derived tumor tissue for p53 analysis). In the absence of a mammalian RP mutant cancer model, the zebrafish RP mutants to date remain the best possible option for molecular studies of RP mutation-driven tumors. That said, we believe our results may still have some important implications for human leukemia. For example, acute myeloid leukemia (AML) has very low rates of *p53* genetic inactivation (similar to the RP mutant MPNSTs) compared to very high rates in many other tumor types [51,52]. Inhibition of GSK-3 with small molecules in AML cells leads to increased differentiation, impaired growth and proliferation, and the induction of apoptosis [53]. Interestingly, the immunomodulating agent lenalidomide, used to treat multiple myeloma, results in the phosphorylation of GSK-3 at the same serine residues that inhibit GSK-3 phosphorylation of MDM2 [54]. This drug has also been reported in 5q-MDS patients to result in increased survival and a reduced risk of transformation to AML [55], raising the possibility that one mechanism of action of lenalidomide is to inhibit GSK-3 phosphorylation of MDM2 and restore a normal p53 response to the acquisition of oncogenic mutations. In sum, we suggest the new mechanisms driving p53 loss that we report here may be useful pathways to target in some cancers.

## Materials and Methods

### Zebrafish strains and maintenance

Zebrafish mutants were created and maintained as described [19,28,56]. Animal experiments were conducted in accordance with the Dutch guidelines for the care and use of laboratory animals, with the approval of the Animal Experimentation Committee (Dier Experimenten Commissie) of the Royal Netherlands Academy of Arts and Sciences (Koninklijke Nederlandse Akademie van Wetenschappen [KNAW] (Protocol # 08.2001).

### *In situ* hybridizations

Performed as previously described using probes against *scl* [31] and *βE1-globin* [57]. At least three independent stains were performed with clutches > 60 embryos. Clutches were stained simultaneously and mutants confirmed afterwards using Sanger sequencing, as previously described [58].

### Morphology

Embryos were sorted by gross phenotype and photographed with a Leica MZ FLIII microscope. At least three independent clutches were analyzed and the expected Mendelian ratio of mutants was always observed. Mutants were confirmed afterwards using Sanger sequencing, as previously described [58].

### Morpholino injections

Embryos were microinjected with ~1ng MO at the one-cell stage. The p53 MO (Gene Tools, Inc. Philomath, OR, USA) is 5’-GCGCCATTGCTTTGCAAGAATTG-3’ and has been previously described [59]. The missense MO sequence is 5'-CATGTTCAACTATGTGTTAGCTTCA-3' (Gene Tools, Inc. Philomath, OR, USA)

### Acridine orange staining and cell counting

Live 1 dpf embryos were incubated in E3-embryo medium + 10μg/mL AO stain (Sigma) in the dark for 30 min. Photos were obtained using a Leica MZ FLIII microscope and cells were blindly counted within a defined area that included the tail starting at the dorsal end of the yolk extension using Image J v1.44 software. At least 8 animals per condition were used for counting. Embryos were genotyped afterwards using Sanger sequencing, as previously described [58].

### O-dianisidine staining

At least 100 embryos per clutch of a mating between two heterozygous hi1034b fish were injected at the one-cell stage with MOs as described above. At 2 dpf they were stained with o-dianisidine (Sigma) as previously described [16]. Scoring of the phenotype severity was done blindly. Embryos were genotyped afterwards using Sanger sequencing as previously described [58].

### TUNEL assays

2 dpf embryos were untreated or exposed to 25 Gy of ionizing radiation. Six hours after irradiation TUNEL assays were performed as previously described and TUNEL-positive cells counted as for AO staining [6].

### Caspase assays

Embryos at 2 dpf were either untreated or subjected to 25 Gy ionizing radiation, and mechanically lysed 6 hours later with a P200 pipette (each sample = 3 embryos per well of a 96-well plate) using 100μL of the western blotting lysis buffer described above. The Caspase-Glo® 2 or 3/7 assay (Promega, Madison, WI, USA) was then performed per the manufacturer’s instructions.

### cDNA synthesis and quantitative PCR

5 embryos per sample were added to 100μL Trizol (Life Technologies, Carlsbad, CA, USA), RNA was isolated and used to make cDNA with the iScript cDNA Synthesis Kit (Bio-Rad, Hercules, CA, USA) per the manufacturer’s instructions. Primers used were as follows: p53 forward 5’- GCTTGTCACAGGGGTCATTT-3’, p53 reverse 5’-ACAAAGGTCCCAGTGGAGTG-3’, GAPDH forward 5’-GGATCTGACAGTCCGTCTTGAGAA-3’, GAPDH reverse 5’- CCATTGAAGTCAGTGGACACAACC, actin forward 5’-GCCCATCTATGAGGGTTACG-3’, actin reverse 5’-GCAAGATTCCATACCCAGGA-3’. Quantitative PCR (qPCR) was performed using iQ SYBR Green Super Mix and a MyiQ Single-Color PCR thermal cycler (Biorad, Hercules, CA, USA). Each experiment was performed in biological triplicate. *p53* mRNA expression in mutants relative to wild types was normalized to *GAPDH* and calculated according to the *C*τ method. Semi-quantitative analysis was performed with a standard PCR method using either p53 or actin primers, the products run on a 1% agarose gel. Statistics were performed with a Student’s t-test.

### Western blotting

Embryos were subject to 25 Gy ionizing radiation and then lysed 6 hours later. 350nM insulin, 10mM Trolox or 100μM LiCl (all from Sigma) was added to the E3 media immediately following the ionizing radiation. For the MG132 (Sigma) experiment, embryo cells were dissociated mechanically with a 200μL pipette tip and added to DMEM media +10% FCS with or without 20μM MG132 for 6 hours at 28°C before lysing the cells. The zebrafish specific p53 antibody and western blotting of zebrafish embryos and MPNST cells has been previously described [20]. For MPNST cells the tumor was dissected, cells mechanically dissociated with a P200 pipette, and plated in a 6 well dish with the indicated concentration of LiCl at 28°C in DMEM + 10% fetal calf serum (Life Technologies, Carlsbad, CA, USA) media overnight. 50μg of total protein from the MPNST cells was used for each sample, measured by Bradford assay (BioRad, Hercules, CA, USA). Other antibodies used at 1:1000 dilution included anti-phospho-AKT (Cell Signaling #9275) and anti-actin (Santa Cruz Biotech, #sc-1616, Santa Cruz, CA, USA). Antibodies used at 1:5000 included donkey anti-goat IgG-HRP (Santa Cruz Biotech, #sc-2020, Santa Cruz, CA, USA) and sheep anti-mouse IgG-HRP (GE Healthcare, #NA931, Little Chalfont, Buckinghamshire, United Kingdom). Quantifications of western blots were performed using Image J v1.44 software. Unless otherwise stated, all embryos subject to western blotting were 2 dpf.

## Supporting Information

S1 Fig RP mutations affect expression of *βE1-globin* but not *scl* A,B) *In situ* hybridizations of 2 dpf embryos measuring the mRNA expression levels of the transcription factor *scl* (A) and the globin gene *βE1-globin* (B). **C)** Representative shots of the dorsal aorta in either wild type of RP mutant embryos stained with probes against *scl* or *βE1-globin*. Note the decrease of the *βE1-globin* expression in the RP mutants compared to the wild type while the expression of *scl* remains unchanged.
(PDF)

S2 Fig Morphology of zebrafish mutants with and without p53 MO. Light microscopy shots of representative wild type (left) or *rpS7* mutant embryos either uninjected (center) or injected with the p53 MO (right) at 1, 2, or 3 dpf. Arrowheads indicate the inflation of the hindbrain vesicle and arrows indicate pericardial edemas, both phenotypes that are rescued by the p53 MO injection.
(PDF)

S3 Fig p53 protein stabilization is impaired in many RP mutant lines. **A)** Semi-quantitative PCR analysis of *p53* mRNA levels in 2 dpf zebrafish embryos carrying mutations in *rpS3a*, *rpL23*, or *rpL36* compared to wild type controls. **B)** Western blot analysis measuring p53 protein in the embryos from (**A**) with or without 25 Gy ionizing radiation (IR). * indicates either a p53 isoform or a degradation product. **C)** Quantification of the ratio of p53:actin bands from the western blots in (**B**).
(PDF)
