# Supplementary figures and images for: Ribosomal Protein Mutations Result in Constitutive p53 Protein Degradation through Impairment of the AKT Pathway
Source: PLoS Genet. 2015 Jul 1;11(7):e1005326. doi: 10.1371/journal.pgen.1005326 (PMC4488577; doi:10.1371/journal.pgen.1005326)

Figure S1

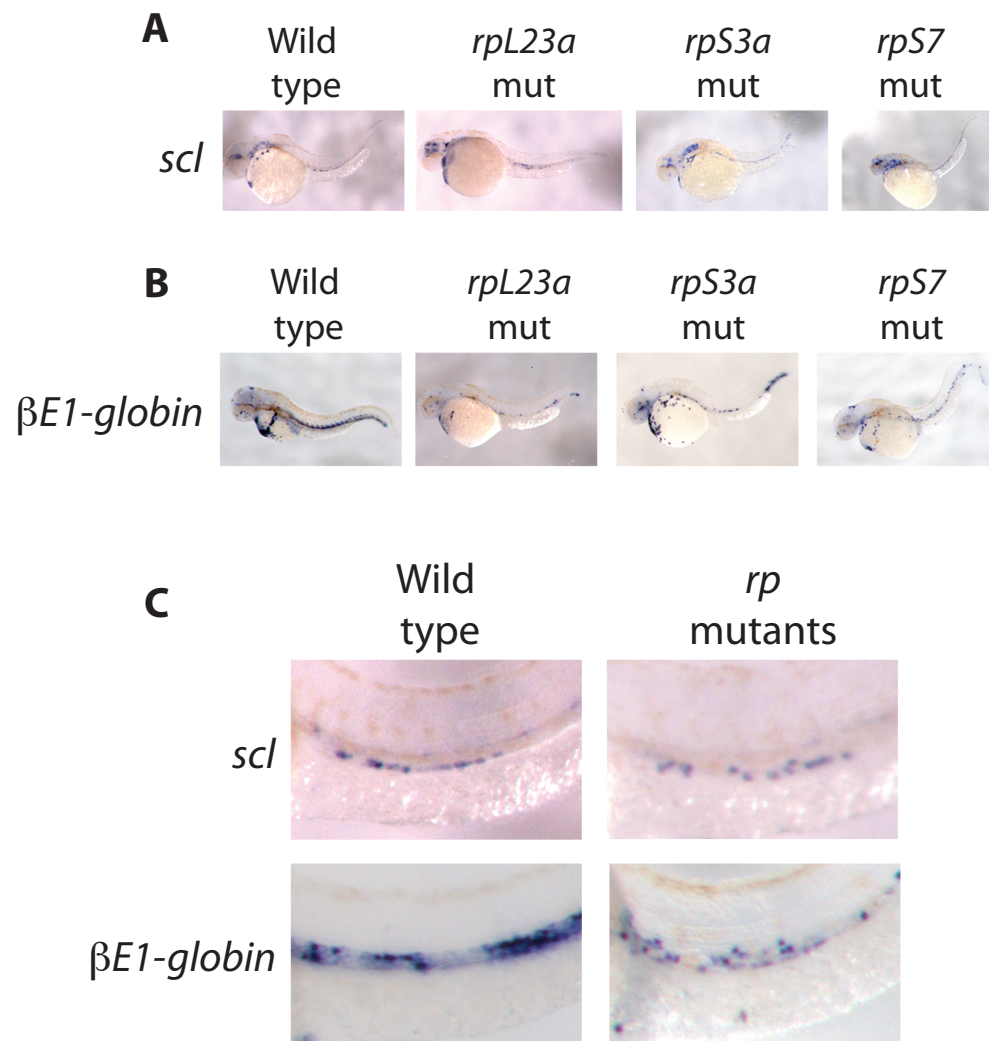

Supplement: S1 Fig — C) Representative shots of the dorsal aorta in either wild type of RP mutant embryos stained with probes against scl or βE1-globin. Note the decrease of the βE1-globin expression in the RP mutants compared to the wild type while the expression of scl remains unchanged. (PDF) [file pgen.1005326.s001.pdf]

Figure S2

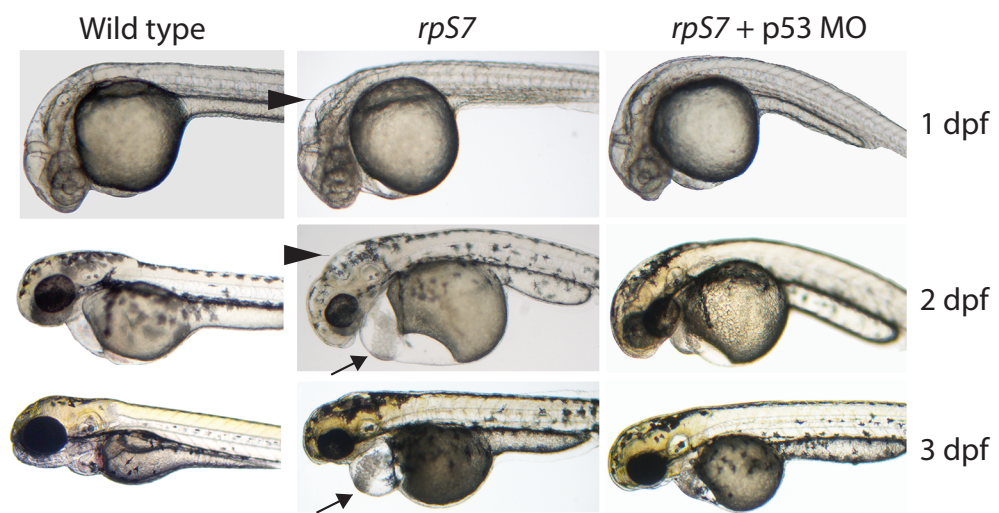

Supplement: S2 Fig — Light microscopy shots of representative wild type (left) or rpS7 mutant embryos either uninjected (center) or injected with the p53 MO (right) at 1, 2, or 3 dpf. Arrowheads indicate the inflation of the hindbrain vesicle and arrows indicate pericardial edemas, both phenotypes that are rescued by the p53 MO injection. (PDF) [file pgen.1005326.s002.pdf]

# Figure S3

**A**

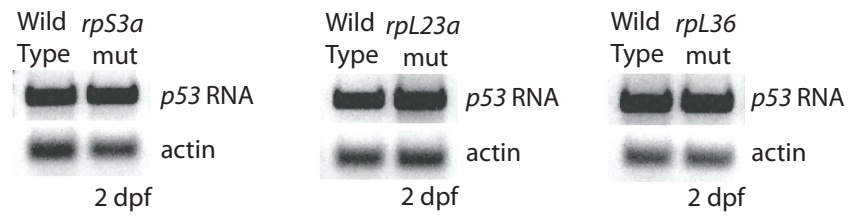

**B**

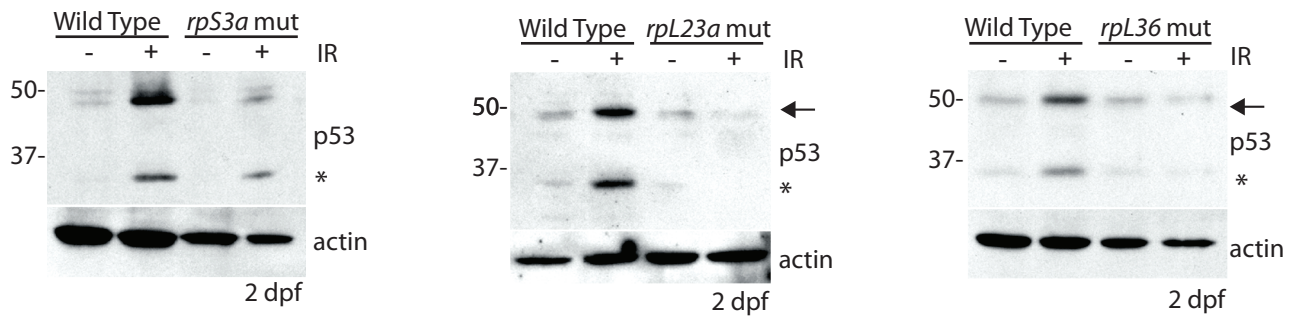

**C**

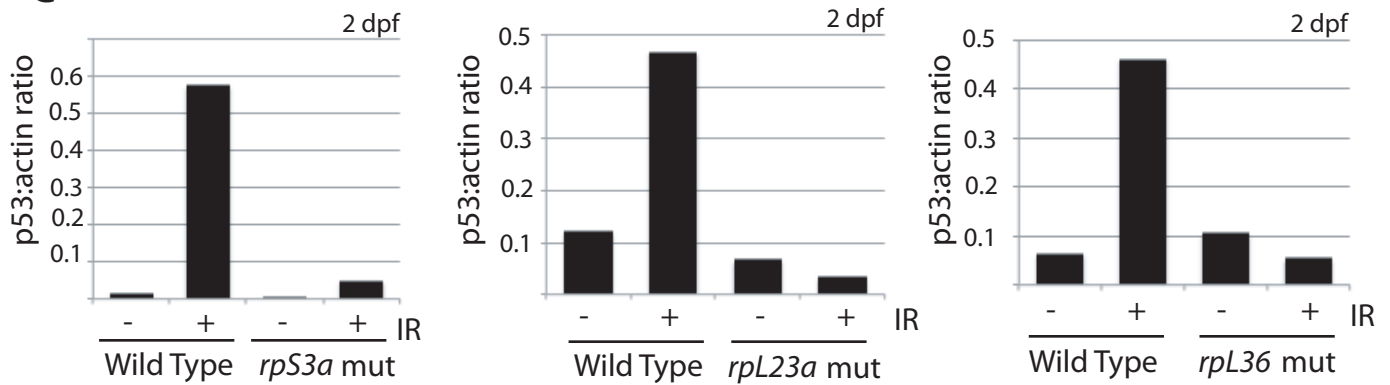

Supplement: S3 Fig — A) Semi-quantitative PCR analysis of p53 mRNA levels in 2 dpf zebrafish embryos carrying mutations in rpS3a, rpL23, or rpL36 compared to wild type controls. B) Western blot analysis measuring p53 protein in the embryos from (A) with or without 25 Gy ionizing radiation (IR). * indicates either a p53 isoform or a degradation product. C) Quantification of the ratio of p53:actin bands from the western blots in (B). (PDF) [file pgen.1005326.s003.pdf]
